# Cataloging the biomedical world of pain through semi-automated curation of molecular interactions

**DOI:** 10.1093/database/bat033

**Published:** 2013-05-23

**Authors:** Daniel G. Jamieson, Phoebe M. Roberts, David L. Robertson, Ben Sidders, Goran Nenadic

**Affiliations:** ^1^Computational and Evolutionary Biology, Faculty of Life Sciences, University of Manchester, UK, M13 9PL, ^2^School of Computer Science, Faculty of Engineering and Physical Sciences, University of Manchester, UK, ^3^Computational Sciences Center of Emphasis, Pfizer, Inc., ^4^Neusentis, Pfizer, Worldwide Research & Development, Cambridge, UK, CB21 6GS, ^5^Manchester Institute of Biotechnology, University of Manchester, UK

## Abstract

The vast collection of biomedical literature and its continued expansion has presented a number of challenges to researchers who require structured findings to stay abreast of and analyze molecular mechanisms relevant to their domain of interest. By structuring literature content into topic-specific machine-readable databases, the aggregate data from multiple articles can be used to infer trends that can be compared and contrasted with similar findings from topic-independent resources. Our study presents a generalized procedure for semi-automatically creating a custom topic-specific molecular interaction database through the use of text mining to assist manual curation. We apply the procedure to capture molecular events that underlie ‘pain’, a complex phenomenon with a large societal burden and unmet medical need. We describe how existing text mining solutions are used to build a pain-specific corpus, extract molecular events from it, add context to the extracted events and assess their relevance. The pain-specific corpus contains 765 692 documents from Medline and PubMed Central, from which we extracted 356 499 unique normalized molecular events, with 261 438 single protein events and 93 271 molecular interactions supplied by BioContext. Event chains are annotated with negation, speculation, anatomy, Gene Ontology terms, mutations, pain and disease relevance, which collectively provide detailed insight into how that event chain is associated with pain. The extracted relations are visualized in a wiki platform (wiki-pain.org) that enables efficient manual curation and exploration of the molecular mechanisms that underlie pain. Curation of 1500 grouped event chains ranked by pain relevance revealed 613 accurately extracted unique molecular interactions that in the future can be used to study the underlying mechanisms involved in pain. Our approach demonstrates that combining existing text mining tools with domain-specific terms and wiki-based visualization can facilitate rapid curation of molecular interactions to create a custom database.

Database URL: •••

## Introduction

One of the largest and most widely used resources of online biomedical literature is the National Library of Medicine’s PubMed (1). PubMed now searches >23 million biomedical records and with other biomedical literature search engines (e.g. Google Scholar, Web of Science and Scopus) is a typical starting point in biomedical knowledge acquisition and information retrieval (IR) (2, 3). For example, a researcher searching for ‘pain’ on PubMed will retrieve 521 141 citations (6 March 2013). This highlights the key problem that arises when the number of relevant unstructured documents from a topical search exceeds the limits of a researcher’s ability to read all (or many) of them. An alternative is to use manually curated resources. Topic-specific curated databases often arise because of unmet needs from existing resources, leading to curation of data not captured by more general sources. They often contain added context that aids the intended users (4–7). Extracting, normalizing and cataloging relevant concepts and facts from free text by dedicated curators make it possible to deal with otherwise unwieldy amounts of information. Accordingly, topic-specific databases that house these findings are rapidly accumulating at an increasing rate (8). Creation of topic-specific databases is well documented (9–11), and there are recurrent themes in the processes used to build high-quality resources. Document triage can be as simple as keyword searches (12–14), but many of these sources have matured enough to shift to sophisticated document classification algorithms (13, 15).

In parallel, there is increasing focus on building tools to help defray the high cost of manual curation (7). There are few databases that are up-to-date with all available relevant information; funding for manual curation is the limiting factor, rather than finding articles to curate. Assisted curation, e.g. through the process of applying text-mining (TM) tools to highlight curatable events, has been repeatedly shown to increase efficiency and reduce curatorial errors (16).

In addition to using TM tools to highlight facts within an article, they can also be used to highlight common facts across articles. We recently reported the recreation of a database of human–HIV-1 protein interactions (17) wherein we proposed a method to group identical interactions mentioned in multiple articles. To increase coverage of unique interactions, it is then only a matter of manually curating selected examples from each group of potentially equivalent interaction mentions. In this system, only one instance of a grouped text mined interaction is required to confirm it as a true positive, enabling rapid validation of molecular interactions derived from TM. Such an approach would acknowledge unique interactions as the primary target of knowledge capture rather than individual mentions, as these are often a valuable feature used by researchers in inferring trends from the overall interactome [e.g. in (18)].

In this study, we explore whether TM tools can be used to create a full-scale disease-specific molecular interaction database from start to finish. Chronic neuropathic pain is an important public health problem, which ∼5–8% of the European population suffers with (19). Current treatment regimens are not universally adequate with only 30–50% of patients reporting an appreciable reduction in pain and improvement in their quality of life using the currently available analgesic drugs such as the gabapentinoids, opioids and selective serotonin reuptake inhibitors such as Carbamazepine (20). In addition, the use of these drugs is often limited by unwanted side effects. There is therefore a significant need for new therapeutics, which requires a better understanding of the mechanisms that mediate chronic pain so that new therapeutic mechanisms might be uncovered. However, there are no existing extensively curated pain-specific molecular interaction databases to facilitate this.

To build a comprehensive pain-related molecular interaction database, we created a pain-specific corpus of biomedical documents using all of the freely accessible literature. From this pain-relevant corpus, we extracted all molecular interactions using the existing BioContext database (http://biocontext.org) constructed from the state-of-the-art in TM. We used existing contexts from this database and added further contexts, such as pain and disease relevancy to interactions, to increase their value to researchers. Finally, we made available the interaction data retrieved to allow manual curation of the grouped results, with the ultimate aim of creating a highly accurate pain-relevant molecular interaction database.

## Methods

### Building a topic-specific corpus

#### Dictionary generation and document retrieval

The first step in generating a full-scale biomedical corpus of documents relevant to pain was to create a pain terms dictionary that could be used to match pain-associated biomedical text. As a basis for the pain terms dictionary, we added terms from an online glossary (21), various pain review articles (22–24) and an in-house term set. Case-sensitive synonyms used in the literature supplemented long forms of pain terms. Ambiguous terms were excluded, as these have been shown to increase false-positive results in IR (25, 26).

**Figure 1.**
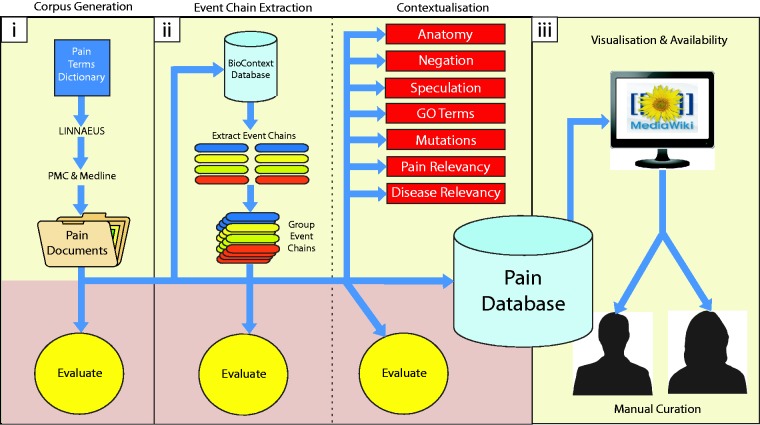
Diagrammatic representation of methodology. Our methodology is divided into three main parts: (i) building a topic-specific corpus and evaluation of document scoring, (ii) data extraction (extracting molecular interactions and adding contexts) and their associated evaluations, (iii) visualization and availability for manual curation of results. Each of these is described in detail within the Methods section.

Dictionary development was iterative, with two rounds of dictionary term review, document retrieval, manual assessment of retrieved documents for absent or ambiguous terms and dictionary modification. After an initial review of retrieved pain documents, we enhanced the pain terms dictionary to improve this procedure. Firstly, we added terms to the dictionary that we flagged as false negatives from the initial corpus evaluation. Secondly, we developed a support tool able to rank strings of tokens based on the proportion of stop words they contained and their size (in number of words). Using this tool, we took the text from the top-ranked 10 000 pain articles to create a list of potential phrases that might be associated with pain. We then manually went through the top terms in this list, adding 33 extra terms to our dictionary. The final dictionary contained 583 terms and 3144 synonyms.

Each term in the dictionary was assigned one of 12 pain-related categories (e.g. pain type, disorder, pain drug, anatomy, condition; see supplementary file 1 for details) to provide more contextual data later in our analysis. Furthermore, a specificity assignment was given to each term to reflect whether the term is specifically relevant to the biomedical research field of pain or if it is a more general term that could apply to other research areas but still has a prominent relevance to pain research. For example, the term ‘neuropathic pain’ was categorized as a pain type and classified as ‘pain specific’. On the other hand, the brain region *locus caeruleus* is not a term synonymous with pain, but it is relevant to pain as an anatomical region involved in the sensation; these are called ‘pain relevant’. In general, terms were classified as pain specific (and assigned a weight of 2) if they were a type of pain disorder, a drug or surgical procedure used to treat pain, a gene with genetic association to pain or a target of a pain drug. Pain-relevant terms (weight of 1) tended to be anatomically or physiologically relevant concepts. The terms and synonyms, including their categorization and pain specificity scores, were inspected by a biologist (RS) with pain expertise.

To match pain-specific terms from our dictionary to biomedical text we used LINNAEUS (27), a named entity recognition tool able to match terms from a predefined dictionary to text. We note that only pain-specific terms were used for document retrieval. We implemented LINNAEUS’s in-built post-processing feature to resolve ambiguity in the results and allow the capture of abbreviations associated with terms in the dictionary. We applied this to all abstracts, titles and MeSH terms in Medline (May 2012 release) and to full text in open access PubMed Central (PMC) (2011 release) that were classified as review or research articles. From herein we refer to our final pain corpus as P1.

#### Document relevance scoring

To quantify the relevance of each retrieved document in the corpus, a document relevance scoring scheme was developed that makes use of both pain-specific and pain-relevant terms, as well as the position of each term’s mention in the document (i.e. title, abstract, MeSH or body). Each pain term matched in a document in P1 was given an individual score based on its textual position (2 if appearing in the title; 1 if appearing in the abstract and in associated MeSH description of the document; 0.25 otherwise) and the pain specificity of the term (2 if pain specific, 1 if pain relevant). These individual scores are then used to determine an overall document relevancy score to pain by summing up the score of all pain terms:

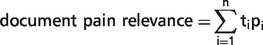

where t_i_ is a term’s pain specificity weight, p_i_ is a term’s position weight and *n* is the number of pain terms in the document. We can similarly calculate pain category relevancy scores (by summing up the score of all pain terms mentioned for each category) and individual pain term relevancy scores (by using all mentions of a given pain term) in each document.

#### Evaluations

To evaluate the effectiveness of our document-scoring scheme, we selected all documents from P1 containing the MeSH term ‘Pain’ and then compared the distribution of document scores for those that had ‘Pain’ as a major MeSH term and those that had ‘Pain’ as a minor MeSH term. We also evaluated individual pain terms matched within 50 documents that had been retrieved in P1. To ensure that we evaluated documents across our pain document scoring range, we randomly selected 10 that scored between 1 and 3 in pain relevancy, 10 between 3 and 10, 10 between 10 and 25, 10 between 25 and 50, and 10 with a score of ≥50.

### Data extraction

#### Extracting molecular interactions

To retrieve the molecular interactions from P1, we used the BioContext database (28). The BioContext database was created from a pipeline of state-of-the-art biomedical TM tools (29, 30) applied to the whole of Medline (May 2011 release) and OA PMC (May 2011 release). Each record in the BioContext database is organized into an event chain originating from a single sentence. Every event chain has a minimum of one and a maximum of three events that were extracted by a union of the two event extraction tools.

Events are categorized into nine types as defined by the GENIA ontology (31, 32), covering protein metabolism (protein catabolism, gene expression and transcription), phosphorylation, localization, binding and regulatory events (positive regulation, negative regulation and regulation). Metabolic events, phosphorylation and localization have a single gene, protein or RNA molecule(s) as their theme (subject), whereas binding events have one or more gene(s), protein(s) or RNA molecule(s) as their theme. Regulatory events are special in that their theme may be a gene, protein, RNA molecule or another event. They are also unique in that they may have a gene, protein, RNA molecule or another event as their cause. Event chains can thus be formed involving multiple molecules and events. For example, ‘CCK-induced expression of fos’ would create an event chain of ‘*CCK* **Positive Regulation** (induced) of **Gene Expression** of *Fos*’. A summary of the events and examples of the event chains that can be formed is provided in supplementary file 2.

The genes, transcripts and proteins that form the themes and causes of each event were extracted using GNAT (33–35) and GeneTUKit (36). Where possible, each mention is then normalized to a species using LINNAEUS (27) and further normalized to an Entrez Gene ID (37) and finally a homologene ID (38).

We took all event chains from BioContext that were extracted from documents present in P1. We then grouped event chains together that contained the same sequence of proteins and events. For example, mentions of the event chain ‘*Ros1* **Positive Regulation** of *NFKB1*’ extracted from multiple sentences and documents were grouped into a single record. Entrez Gene IDs were used to group proteins instead of gene symbols to prevent erroneous grouping caused by naming ambiguity.

To group event chains involving a binding event with two molecules we had to resolve instances where the order of the proteins varied across analogous event chains. For example, one event chain may be directed as, ‘Binding of *CD44* and *MMP9*’, whereas another may vary as such, ‘**Binding** of *MMP9* and *CD44*’. Because the order of proteins in binding events does not infer any functional characteristic of the data (binding of CD44 and MMP9 is the same), classing these as separate unique event chains when grouping would be erroneous. Thus, we rearranged binding proteins numerically using Entrez Gene IDs when proteins were normalized or alphabetically otherwise.

During the grouping of each event chain, we recorded the total frequency of that event chain and the number of documents that each event chain was reported in. We also stored the number of molecules involved in each event chain. This enabled us to define molecular interactions as those event chains containing two proteins, genes or RNA molecules. Those containing only a single molecule are referred to as single events. TM confidence scores provided by BioContext for each grouped event chain were determined by taking the highest confidence score from the associated event chains used in the grouping.

#### Molecular interaction extraction evaluation

The individual tools used in BioContext to create the event chains used in this study have already been extensively evaluated (28). We used the results from the final manual curation step (see below) for direct evaluation of grouped molecular interactions.

Pain-relevant interactions extracted for this study should be enriched for proteins previously linked to pain. Therefore, we also undertook an enrichment analysis, comparing event chains retrieved from P1 with a set of interactions derived from a random set of documents for the presence of known pain-associated proteins. The genes/proteins used as a gold standard pain set were taken from the Pain Genes DB (39). This set contained 297 unique manually curated genes. We measured how many unique and total mentions of genes were present in our event chains (both single events and molecular interactions). The generic set of event chains was formed from the same number of randomly selected Medline and PMC documents as P1, but which were not present in P1. Event chains from this random document set (referred to as R1) were then extracted from the BioContext database and grouped using the same procedure as used in constructing the event chains from P1. Unique and total mentions of pain genes present in R1 event chains were then determined. Fisher’s exact test was used to statistically evaluate whether P1 was enriched for pain genes in event chains in contrast to R1.

#### Adding context to molecular interactions

As well as the species context for proteins, BioContext also contains anatomy, negation and speculation context for each event chain. Anatomical mentions in the text (such as ‘peripheral nerve’ or ‘spinal cord’) and cell-line mentions used as proxies for anatomical locations were extracted using GETM (40). These anatomy mentions were, where possible, mapped to events to provide details on the anatomical location of an event.

Negation and speculation detection was provided for each event in BioContext using a modified version of Negmole (41). Instances of negation (e.g. ‘Lep did not bind to Obsty1’) and speculation (e.g. ‘Lep maybe binds to Obsty1’) are extracted and annotated on the resulting event chain (i.e. ‘[Negative] **Binding** of *Lep* and *Obsty1*’ or ‘[Speculative] **Binding** of *Lep* and *Obsty1*’).

We additionally provide four other contextual features: associated gene ontology (GO) terms and mutations, and pain and disease relevance scores.

GO terms (42) and their overarching GO Slim terms (43, 44) were added to normalized proteins where feasible to provide more functional information on proteins involved in each event chain. This was achieved using the publicly available Gene2Go mapping of Entrez Gene IDs to GO IDs available on the National Center for Biotechnology Information FTP service (45).

Point mutation context was added to proteins in event chains by using MutationFinder to match and normalize mutation instances in the text (46). MutationFinder was run only on sentences that were the source of each event chain in our pain set. However, because MutationFinder is unable to link mutations to any associated protein mentions in the text, we designed and implemented our own system to do this. We formulated a number of priority-ranked regular expressions to match commonly occurring textual patterns, e.g. ‘<protein> - <mutation>’ or ‘<mutation> for the <protein>”. Our system also allowed individual proteins to match multiple mutations, e.g. ‘mutations <mutation A>, <mutation B> and <mutation C> for <protein>’. The regular expressions used are provided at wiki-pain.org/downloads.

We designed a novel method to calculate the relevance of each pain term to an event chain in a document (note that this is distinct from the document relevance method described above). The score ranges from 5 to 100 and reflects the likelihood that a pain term is relevant to a given event chain. The algorithm uses the document sections in which the pain term and the event chain are mentioned (i.e. title, abstract, MeSH and body), whether they co-occur in a sentence, and where appropriate the distance between the two and the order that each is presented. For example, a pain term mentioned in the same sentence as an event chain receives a score of 75–100. Pain terms matched in different sections to a given event chain are given lower relevancy scores. We were then able to produce an overall relevancy score to pain for an event chain using individual relevance scores of each pain term >50 to that event chain and weighting by pain term specificity. A more detailed description of the scoring calculation with examples is provided in supplementary file 3.

The final context added to our event chains is disease relevancy. Pain, although often considered a disease in itself, is commonly related to symptoms of a whole host of other diseases. To allow researchers to explore these trends in relation to interactions, we matched disease terms from an in-house disease lexicon (containing 4861 terms with 205 373 case-sensitive synonyms) to P1 using LINNAEUS (27). We then adopted the same method used in the pain relevancy scoring to calculate the relevancy of each event chain to each disease term match and from these the overall disease relevancy of each grouped event chain (without the term weighting).

#### Context evaluations

We did not repeat the existing evaluations performed in BioContext (28) for anatomy, negation and speculation contexts. Similarly, mutation detection and normalization had also been previously evaluated for MutationFinder (46). However, to evaluate the mutation to protein linking method, we selected 100 event chains that matched at least one mutation in the original sentence used to extract the data. As well as noting true positives, false positives and false negatives we marked true negatives defined as those mutation mentions correctly left unlinked to a protein in an event chain.

To assess the event chain relevancy scoring system to individual pain terms, we randomly selected 100 linked event chains and pain terms that scored >50 and another 100 that scored <50. A true positive was given if the term bared some notable relevance to the event chain in question, whether a direct or indirect association.

Our disease relevancy evaluation first assessed the disease term matching performed by LINNAEUS in 50 randomly selected documents that had matched at least one disease term. As above for the pain relevancy evaluation, we selected 100 linked event chains and disease terms that scored >50 and another 100 that scored <50 for disease term to event chain relevance evaluation.

### Availability and visualization for manual curation

To visualize and make our data available to researchers, the MediaWiki (version 1.19) framework was used, as this platform has been successfully used in other database representations (47). The primary use of this system (available at wiki-pain.org) is to support curation of pain-related molecular interactions by providing an infrastructure for assessing data proposed by TM as described above. We built wiki-pain.org using the MediaWiki API to automatically upload pages constructed from our databases (48).

As a pilot, we performed manual curation on the top 1500 grouped molecular interactions (ordered by overall pain relevancy scores) involving human, mouse or rat proteins and excluding self-interactions, marking each as either a true positive or false positive. The task was spread across three curators, 500 assigned to D.J., 500 to B.S. and a further 500 to three biologists (VA, LR and MK).

Traditional evaluations of events and their protein constituents have focused on selecting a set of articles and scanning the text for requisitioned data and comparing this against the data retrieved (49). As grouped interactions can be formed from a number of different documents, to fully evaluate even a small number of these using a traditional evaluation would require masses of documents to be assessed. Thus, we chose to evaluate grouped event chains by selecting individual mentions of an event chain ordered by TM confidence and their associated sentences (and documents if needed for further verification) and used these to determine whether an overall grouped event chain was a true positive or a false positive. We required only one correct individual event chain of a group to determine it as an overall true positive. While this form of evaluation requires much less time spent reading each full article, we recognize that as a result we do not measure the frequency of false-negative instances.

We evaluated each individual event chain using the stringent form of evaluation as described previously (17). This evaluation requires the full event chain including all of its participants to have been extracted and normalized accurately to their correct species and Entrez Gene ID to be classed as a true positive.

To assess the quality of our manual curation, we determined the inter- and intra-annotator agreement by one curator blindly recurating 50 randomly selected molecular interactions previously curated by that curator (intra) and 50 randomly selected molecular interactions previously curated by other curators (inter). Furthermore, to assess how many individual mentions a curator needed to curate to determine a grouped molecular interaction as a true positive, we sampled 100 random true-positive grouped interactions that contained at least five mentions of that interaction from our curated data. We then assessed the proportion of individual mentions that were correct in each grouped molecular interaction.

## Results and Discussion

### Building a topic-specific corpus

#### Pain terms dictionary

**Figure 2.**
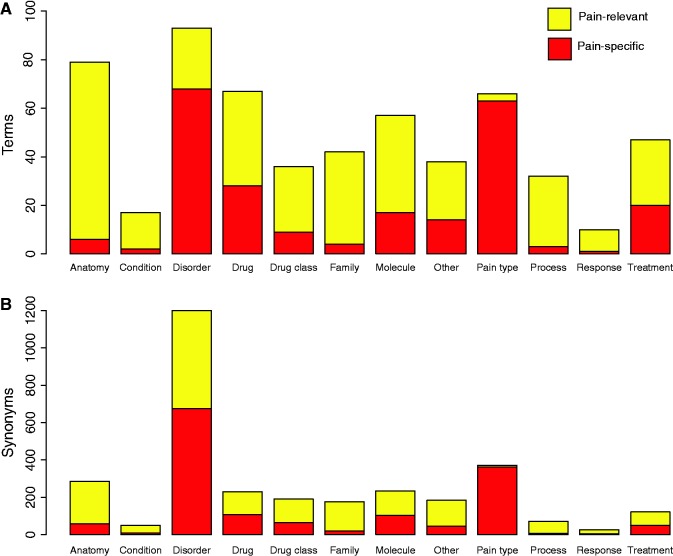
Pain dictionary summary statistics. (**A**) Represents the numbers of pain-specific and pain-relevant terms in the pain dictionary for each category of pain term. (**B**) Represents the numbers of pain-specific and pain-relevant synonyms in the dictionary for each category of pain term.

Figure 2 displays the final counts of pain-specific and pain-relevant terms and synonyms for the 12 categories of pain terms. In total there were 583 terms (235 pain specific and 348 pain relevant) and 3144 case-sensitive synonyms (1506 pain specific and 1638 pain relevant). We note that there are high proportions of pain-specific ‘disorder’ and ‘pain type’ pain terms. We note that while in this study the pain terms dictionary created was sufficient for building an accurate corpus of pain documents, future implementations of our approach in other biomedical fields may be better suited to using existing ontologies and controlled vocabularies for example, SNOWMED CT (50).

#### Document retrieval

**Figure 3.**
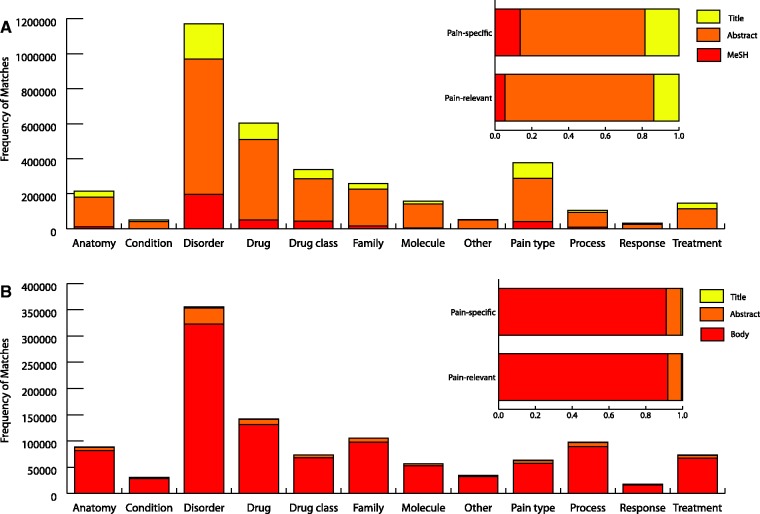
Pain term matches. Pain term matches from Medline (**A**) and open access PMC documents (**B**) in each type of document section across the 12 pain term categories are displayed. The overall percentage of pain-specific and pain-relevant terms from Medline and open access PMC documents are shown for each type of document section. ‘Body’ represents full text excluding abstracts and titles. MeSH refers to textual document tags used by PubMed articles in indexing.

The total number of matches in different document sections of pain-specific and pain-relevant terms for each pain term category is shown in Figure 3. There were matches of pain-specific and pain-relevant terms in all of the 12 categories with a large proportion coming from disorder terms. Altogether there were 4 645 861 pain term matches, 2 548 287 pain specific and 2 097 574 pain relevant. Matches of pain-specific and pain-relevant terms were made across each type of document section in P1 with a large proportion being made in the abstracts. However, while this distribution of terms across different textual sections is representative of our corpus, we would expect that the proportion of terms found in the body of a document would be far greater had we had access to full text not available in our Medline data set. For instance, if we exclude Medline documents from our sectional analysis, 91% of matches are found in the body of the article.

Table 1 displays the top 10 reported pain terms in P1, ordered by the number of documents that they were reported in. Nine out of ten terms were pain specific and they accounted for roughly 25% of all matches. From our pain-specific matches, there were 765 692 documents (732 826 Medline and 32 866 PMC) that matched at least one term. Of the 32 866 PMC open access documents that were part of P1, these composed roughly 17% of the entire PMC open access corpus in comparison with 7% of Medline from 732 826 documents. It is likely that this disparity was caused by a greater availability of text accessible for matching terms from our pain dictionary in full text documents. This perhaps indicates that many documents that are pain relevant in Medline have been missed, as we have not had access to terms located in associated full text.

**Table 1.** Top reported pain terms in P1

Pain Term	Category	Pain Specific	Frequency	Documents
Pain	Disorder	Yes	627 644	247 312
Anaesthesia	Pain type	Yes	190 376	115 614
Analgesic	Drug class	Yes	112 703	61 223
Headache	Disorder	Yes	118 956	50 249
Brain haemorrhage	Disorder	No	85 702	45 214
Opioid	Drug class	Yes	77 921	33 486
Morphine	Drug	Yes	119 985	33 337
Analgesia	Pain type	Yes	64 777	31 982
Palliative	Treatment	Yes	51 401	27 536
Abdominal pains	Pain type	Yes	33 916	25 062

‘Pain term’ refers to the individual pain term and all its synonyms. Pain terms are pain specific (yes) or pain relevant (no). Pain term ‘categories’ are defined in supplementary file 1. ‘Frequency’ refers to the total number of times that that term was mentioned. ‘Documents’ refers to the number of documents that that term was mentioned in.

**Figure 4.**
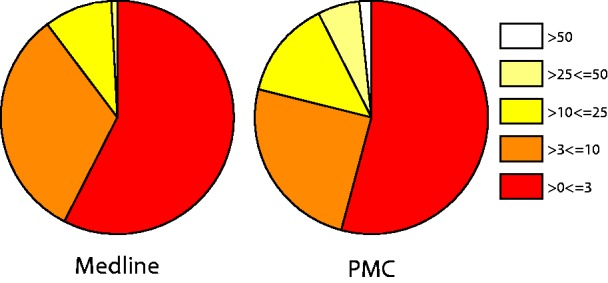
Document pain relevancy scores. Pie charts represent the overall pain scores for Medline (abstracts and titles).

The overall pain document relevancy scores are summarized in Figure 4. The analysis of this scoring scheme showed that documents with the MeSH term ‘Pain’ as a major term scored significantly higher than those that had ‘Pain’ as a minor MeSH term when using a Wilcoxon/Krustai–Wallis test (Z = −49.326 and *P* < 0.001). Further information is provided in supplementary file 4. This initial evaluation shows that as well as being able to retrieve pain documents, we can also differentiate between these in terms of their overall relevance to pain using our scoring system. As well as this overall pain relevancy score, the pain category and individual pain term scores allow for exploration of specific aspects of pain. Indeed, our evaluation of the pain terms present in 50 reported pain documents showed 100% precision and 89.6% recall (Table 7), highlighting that we have been able to extract individual pain concepts with high accuracy.

However, we note from Figure 4 that documents where full text was used scored higher than articles with only abstracts and titles available, highlighting a potential issue in our scoring method when using documents of varying textual lengths. At present, our method partially addresses this by scoring terms matched in the body of an article with 0.25, in comparison to terms scored with 1 in the abstract and 2 in the title. However, in future corpus generation, the section weights could be adjusted to produce a score that does not bias full text articles into being scored higher.

### Data extraction

#### Event chains

In total there were 1 578 654 event chains from the BioContext database present in P1. After grouping these event chains, there were 356 499 unique event chains, with 261 438 single events, 93 271 containing two participants (i.e. molecular interactions) and 1790 involving more than two participants. Table 2 shows the frequencies of single events, molecular interactions and interactions with more than two participants involving proteins normalized to humans, mice, rats and other species. Human, mouse and rat proteins incorporated 44% of unique single events and 37% of unique molecular interactions with the other proteins in event chains being normalized to 1230 different species. As humans, mice and rats are the model animal species studied in pain molecular research, these results show that there are large amounts of useful data available for curating a pain-relevant molecular interaction database.

**Table 2.** Event chains from P1

Involving only	Single events	Molecular interactions	More than two participants	Total
Human proteins	45 731	14 568	262	60 561
Mice proteins	41 671	12 956	230	54 857
Rat proteins	26 736	7369	132	34 237
Other proteins	147 300	58 378	1166	206 844
Total	261 438	93 271	1790	356 499

Event chains are shown for those involving only human, mice, rat and other proteins as their cause and/or theme. Event chains are divided into single events, molecular interactions (i.e. those containing two participants) and event chains with more than two participants. Total numbers of events chains by number of participants and by proteins involved are displayed.

Table 3 shows the number of grouped event chains involving events of protein metabolism, binding, localization, phosphorylation and regulation. We found large numbers of regulatory and binding events involved in all types of event chains and high numbers of gene expression events in single events.

**Table 3.** Event types involved in event chains

Event type	Single events	Molecular interactions	More than two participants
Binding	33 358	37 291 (37 315)	897 (919)
Gene expression	78 255	12 223 (12482)	95
Transcription	12 158	1238	10
Localization	27 329	5355 (5368)	50
Phosphorylation	7360	1782 (1784)	37
Protein catabolism	5296	467	6
Positive regulation	69 846 (75 064)	32 222 (35 740)	1174 (1650)
Negative regulation	52 754 (54 729)	13 698 (14 870)	541 (624)
Regulation	41 137 (42 422)	19 271 (19 783)	468 (551)

Non-redundant frequencies of single events, molecular interactions (i.e. those containing two participants) and event chains containing more than two participants are displayed for each of the nine categories of events used by the event extractors. The numbers in brackets represent the total number of occurrences of that event type where some events have duplicate (redundant) event types, e.g. ‘**positive regulation** of **positive regulation** of *protein A*’.

**Figure 5.**
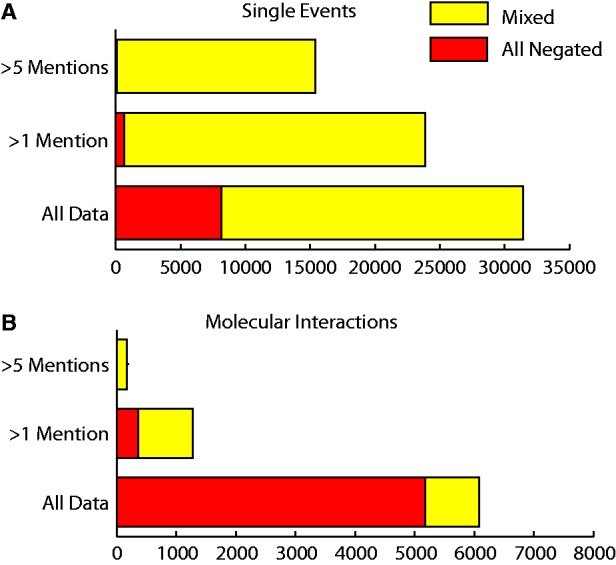
Number of negated event chains. ‘Mixed’ refers to event chains that have been mentioned both negatively and positively. ‘All negated’ refers to the number of event chains that are only mentioned negatively. Proportions of mixed and negated data are shown for all molecular interactions and single events that have been mentioned more than once or more than five times.

In total there were 37 628 grouped event chains that were reported negatively at least once. Of these, 24 142 potentially represented contradictions with some mentions of a grouped event chain being reported negatively and others positively. Of those event chains that were reported more than once, there were only 25% (369/1457) that were reported entirely negatively (Figure 5). A total of 31 275 (26 268 single events and 4909 molecular interactions) grouped event chains were reported speculatively at least once. Of those event chains that had been reported more than once, 277/1207 molecular interactions and 382/20 931 single events were reported entirely speculatively.

From the 356 499 grouped events chain, 172 294 were mapped to at least one anatomical region. Table 4 exhibits the top 10 anatomical regions (of 2774 total) associated with event chains retrieved from P1; these made up ∼27% of all anatomical mentions in our pain data set. We note high numbers of immune anatomical structures, which is not unexpected with pain-related data (22).

**Table 4.** Top 10 anatomical regions associated with event chains

Name	Frequency
Neurons	37 666
Plasma	36 969
Brain	31 775
Blood	19 291
T cells	16 092
Liver	15 650
Spinal Cord	14 453
Macrophage	13 409
Neuronal	12 368
Nerve	11 355
Total	761 990

Anatomy terms are extracted using GETM.

From sentences used to extract event chains in P1, we were able to map 2997 mutations to proteins involved in single events and 721 mutations to proteins involved in molecular interactions.

Table 5 provides an overview of the overall pain relevancy scores calculated for each unique event chain in our data set involving human, mouse or rat proteins (the most commonly studied animal models in pain research) and excluding self-interactions (e.g. ‘**Binding** of *Tprv1* and *Tprv1*’). The mean overall pain relevancy score for these was 0.33, with a median of 0.15 and standard deviation of 0.64. There were 25 593 medium pain ranked (between 0 and 1 in overall pain relevancy) and 2646 highly relevant (>1 in overall pain relevancy) unique pain molecular interactions.

**Table 5.** Overview of overall pain relevancy scores for unique event chains involving human, mouse or rat proteins and excluding self-interactions

Pain relevancy score	single events	Molecular interactions	More than two participants	Total
Low (0)	22 623	9240	191	32 054
Medium (>0,≤1)	62 640	25 593	520	88 753
High (>1)	28 875	2646	42	31 563

We show the frequency of unique single events, molecular interactions (i.e. two participants) and event chains with more than two participants with a low (0), medium (>0, ≤1) or high (>1) overall pain relevancy score.

In total we matched 6 792 990 disease terms in 618 487 documents from P1, allowing 3 041 109 disease terms to be mapped to 1 402 560 event chains. Table 6 displays the top diseases associated with P1 documents containing event chains. While generic classes of disease terms, such as ‘disease’, ‘injury’ and ‘inflammation’, featured in the top 10, there were also high numbers of ‘diabetes-’, ‘pain-’, ‘depression-’, ‘cancer-’ and ‘HIV-’associated event chains. We note that these have a large neuropathic pain component.

**Table 6.** Top diseases associated with documents containing event data

Disease name	Disease term mentions
Disease	135 367
Pain	122 233
Cancer	117 041
Inflammation	101 059
Injury	59 237
Infection	57 481
Diabetes mellitus	50 705
Stress	41 056
Depression	39 762
AIDS or HIV infection	30 872
Total	3 041 109

Here we report the total number of disease term mentions in documents that contain at least one event chain.

#### Data extraction evaluations

Table 7 displays the results for all of the new evaluations of methods used in this study.

**Table 7.** Evaluations of TM software used

Tool	Data	True positives	False positives	True negatives	False negatives	True negative Rate	Precision	Recall	Accuracy	F score
Pain terms (LINNAEUS)	50 Documents	3803	0	N/A	443	N/A	100	89.6	N/A	94.5
Mutation to protein linker	100 Event chains	36	1	109	14	99.1	97.3	72	90.60	82.7
Pain relevancy (>50 confidence)	100 Event chains	78	22	N/A	N/A	N/A	78 (92 expected)	N/A	N/A	N/A
Pain relevancy (≤50 confidence)	100 event chains	39	61	N/A	N/A	N/A	39 (20 expected)	N/A	N/A	N/A
Disease terms (LINNAEUS)	25 Documents	345	16	N/A	15	N/A	95.6	95.8	N/A	95.7
Disease relevancy (>50 confidence)	100 Event chains	84	16	N/A	N/A	N/A	84 (88 expected)	N/A	N/A	N/A
Disease relevancy (≤50 confidence)	100 Event chains	30	70	N/A	N/A	N/A	30 (13 expected)	N/A	N/A	N/A

For each tool evaluated we display a summary of the data used in the evaluation (either documents or event chains), and the frequencies of true positives, false positives, false negatives and true negatives for each tool wherever possible. From the true positives, false positives, false negatives and true negatives we calculated the true-negative rate, precision, recall, accuracy and F score of each tool where applicable. In pain and disease relevancy we also note the expected precision calculated from the average relevancy score of each term in the respective evaluation.

Our mutation-to-protein linker (of co-occurring mentions in sentences) extension for MutationFinder showed precision of 97.3% and recall of 72% to give an F score of 82.7%. The mutation-to-protein linker also showed a 99.1% true-negative rate to give an accuracy of 90.6%. Improvements to recall can be facilitated by extending our library of regular expressions. At present our tool is only able to normalize proteins to mutations that are both denoted in the same sentence; however, in our analysis we noted a large number of proteins associated with mutations that were defined outside of the sentence. This limitation, as well as the accuracy involved in extracting the original event chain and the mutation mention itself, is important to consider when using such data.

In the evaluation of pain terms relevant to event chains with scores >50, we judged 78/100 as relevant. These results are lower than the predicted 92/100 taken from the average relevancy score across the 100 event chains evaluated. We noted that ‘molecule’ and ‘family’ category pain terms were more likely to be irrelevant to an event chain when mentioned outside of the sentence the event chain was denoted in. By contrast, the evaluation of pain terms relevant to event chains with scores <50 showed that 39/100 relevant pain term–event chain pairs, whereas the expected value was 20/100. The higher than expected number was mainly caused by ‘disorder’ pain terms that, although mentioned in distant sentences to the event chain, were still perceivably relevant.

Judging from 25 documents, our disease term matching showed a precision, recall and F score of 96%. Our evaluation of the linking of these terms to event chains in which relevancy scores were >50 showed 84/100 relevant disease term–event chain pairs. The average predicated relevance score across each linked disease term–event chain pair was 88, indicating that our high relevance predictions were fairly accurate. However, in the evaluation of relevancy of disease terms–event chain pairs with scores <50, we found 30/100 disease terms to be relevant compared with the 13/100 predicted. As with our low pain relevancy evaluation findings, we found that disease terms could still be relevant to an event chain even if they were mentioned in paragraphs and sentences at some distance from the event chain in the text. These issues for both pain and disease relevancy can be resolved by adjusting each approach to more closely reflect the likelihood of actual disease or pain relevancy.

Because we are using event chains directly from BioContext, we expect that event extraction precision and recall will be consistent with previously reported ones (17, 28). Indeed, benchmarking against a small manually curated gold standard of five full text documents reported similar precision, recall and F score of 35%, 58% and 44%, respectively. A detailed analysis is available in supplementary file 5. A comparison of TM data against existing generic manually curated databases is difficult as there are no extensive pain-focused resources that can be used directly. Instead, we have explored the intersection between our TM results and iRefIndex, a large generic molecular interaction database containing interactions from numerous species sourced from various individual manually curated databases (51). As expected, the overlap is not significant (only 21 interactions) given the difference in the criteria used to extract and represent the data between data sets. We have provided this analysis in supplementary file 6.

To determine whether genes known to be related to pain were enriched in the P1 extracted events, an enrichment analysis was performed (Table 8). In total 280/297 genes in the Pain Genes DB were mentioned in at least one of our event chains. These genes were mentioned in 4.54% of event chains in P1, which was more than double the 2.14% found in R1. Fisher’s exact test confirmed P1 to be enriched for these genes with an odds ratio of 2.18 and a highly significant *P* value (<2.2e-16), suggesting that the overall data set of molecular events recovered from our corpus are relevant to pain.

**Table 8.** Pain genes enrichment analysis

Corpus	Event chains mentioning a pain gene	Event chains not mentioning a pain gene	Total event chains	% of event chains with a pain gene
P1	71 685	1 506 969	1 578 654	4.54
R1	47 998	2 196 618	2 244 616	2.14

P1 represents the pain corpus and R1 represents the randomly generated generic corpus. We show frequencies of event chains mentioning a gene from the Pain Gene DB for each corpus and event chains not mentioning a gene from the Pain Gene DB. We also display total event chains for each corpus and the percentage of event chains that contain genes from the Pain Gene DB. Fisher’s exact test showed significant enrichment of pain genes within P1, having an odds ratio of 2.177008 with a *P*-value <2.2e-16.

Of the 17 genes from the Pain Genes DB that were not mentioned in event chains from P1, 15 were mentioned in BioContext event chains extracted from Medline and PMC documents there were not in P1. To determine why these genes were found outside of P1, we selected five random articles that mentioned one of these genes in an event chain for each of the 15 genes (75 articles in total). Of the 75 articles, they were all pain irrelevant, with a small number mentioning pain-relevant terms (e.g. GABA). Four of the genes did not have a correctly reported mention in the articles sampled, with the majority of the errors coming from erroneous gene name normalization.

### Availability and visualization for manual curation

#### Data availability

**Figure 6.**
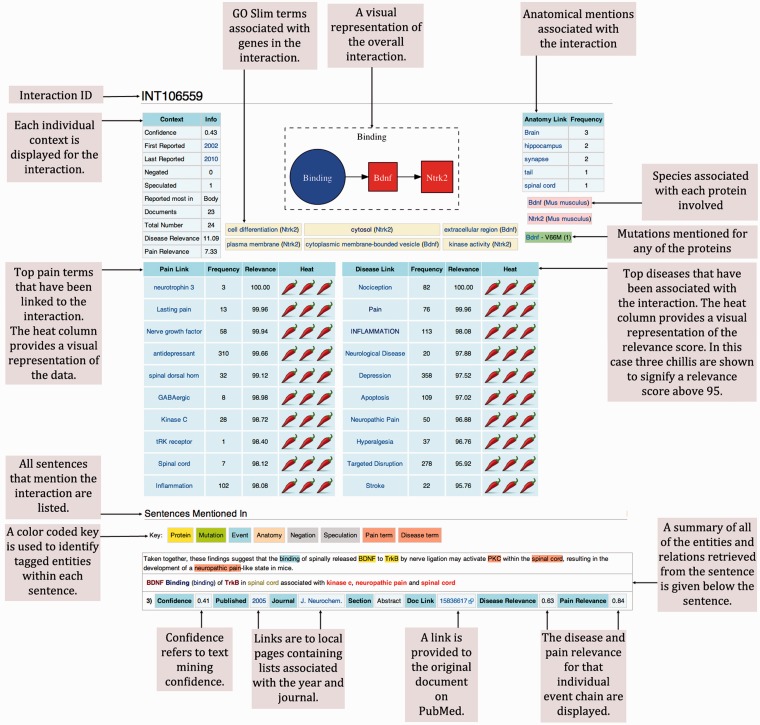
Example of a typical molecular interaction in wiki-pain.org. We have removed the page borders that are typical of a Mediawiki interface and annotated each region of the page that we have designed and is novel. All ‘INT’ pages on wiki-pain.org follow the same framework including single events and event chains containing more than two participants. The specific page shown can be viewed by searching ‘INT106559’ on wiki-pain.org.

We uploaded the data retrieved from our investigation onto wiki-pain.org. At the core of the wiki are the ‘INT’ pages (Figure 6) used to display each grouped event chain relevant to pain. Within each page summary, contextual data are displayed at the top, providing a visualisation of that event chain’s relevance to pain. Beneath the summary information are the sentences where the data was extracted from highlighting entities extracted using a color-coded key. Each sentence then has its own summary, providing links back to its original source among other useful contexts that can be used for further investigation.

The INT pages are named using INT IDs to enable linking across the site. Most INT links stem from summary tables created to help guide users to the most relevant information. For example, the entry page on the wiki contains summary tables of all interactions and single events in the database ordered by their overall relevance to pain. Other summary tables can be found on gene pages, journal pages, event-type pages, disease-term pages, etc., linking interactions specific to page type, e.g. on the G:60628 (CXCR4) page, only event chains mentioning this gene are displayed.

#### Manually curated data

The manual curation of the top 1500 grouped molecular interactions showed 613 true positives and 887 false positives. This means that grouped molecular interactions have a precision of 40.87% before they have been curated. However, if we set a cutoff of 50% for the TM confidence (coming from BioContext), our precision more than doubled to 84.17% (117 true positives and 22 false positives). We also found that unique interactions mentioned in more than one document were more likely to be a true positive, with precision of 59.71% (252 true positives and 170 false positives) in comparison with 33.48% (361 true positives and 717 false positives) mentioned in only one document. Therefore, for supporting curation, it makes sense to prioritize using high-confidence interactions only.

Overall, the 613 true positives included 487 different genes, with 161 human proteins, 170 mouse proteins and 156 rat proteins. These genes could be grouped into 351 homologues (by using their homologene IDs), indicating a variety of proteins in the curated data and not simply those proteins synonymous between species. Table 9 shows the top 10 homologues ordered by frequency of unique molecular interactions that each is involved in. We also found 90/276 homologues and 61/297 of the previously identified pain-relevant genes from the Pain Genes DB in our manually curated data set. These results indicate that we have identified 261 additional homologous sets of genes that could potentially be associated with pain, including 426 specific genes.

**Table 9.** Top 10 homologues appearing in our manually curated data

Homologue ID	Symbol	Frequency
1876	NGF	53
37368	OPRM1	50
723	POMC	45
12920	TRPV1	44
88337	CALCB	40
4528	PENK	39
502	IL6	27
599	CRH	22
496	TNF	19
4537	PNOC	16

These have been ranked by frequency of unique molecular interactions that each homologue is involved in, in our manually curated data. Homologue ID refers to the ID used by NCBI homologene database (http://www.ncbi.nlm.nih.gov/homologene).

Of the false positives, we noted commonly occurring causes such as incorrect protein normalization to Entrez Gene IDs and event mismatches. We also noted a large number of false positives caused by abbreviations tagged as proteins that were in fact other types of entities (e.g. ‘long-term potentiation (LTP)’ that was erroneously normalized to the ‘LTP gene’). This problem can be resolved by better integration of biomedical entity-tagging tools to filter out instances of data by pre- or post-processing that which had been previously defined as another entity type.

To determine how many of the true-positive molecular interactions were present in existing manually curated databases, we checked protein pairs from our data against MiMi (52), a large online database incorporating multiple data sources [BIND (53), HPRD (54), IntAct (55), etc.], through the MiMi API. In total we retrieved 59 protein pairs in MiMi from 505 present in our data set, indicating that the majority of our true positive (curated) data has yet to have been incorporated into the existing curated databases.

In the assessment of the proportion of individual mentions of a grouped event chain that were correct, we removed 12 grouped interactions from the analysis that had previously been reported as true positives and after review were determined to be false positives. From the remaining 88 grouped interactions, we found 335 correctly identified individual mentions against 105 incorrectly identified mentions, highlighting on average three true positives in the top five mentions of a grouped interaction. These results show that for grouped interactions a high proportion of the top five individual mentions are correct and therefore curators do not need to spend added time curating each and every individual mention when the overall grouped molecular interaction is a true positive.

Having manually curated 4% of all extracted interactions, we sought to infer what proportion of the uncurated interactions were likely to be true positives. The TM confidence score for each interaction (deduced by BioContext) separates the true from false positives relatively well. The true- and false-positive interactions have a mean confidence score of 0.3 and 0.1, respectively, and are significantly different (*P* < 0.0001). We therefore fit a generalized linear model following a binomial distribution with a logit link function to the confidence scores from the curated data, so that we can assign a probability of being correct to the remaining 36 732 grouped interactions. We found that interactions with a TM confidence score >28% were likely to be true positives. Using this measure, we can predict that 5816 of the remaining interactions are more likely to be true positives than false positives (see supplementary file 7 for further details on these calculations). For this study, it took on average one working day for a curator to curate 250 molecular interactions. Therefore, we can assume that it would take one curator a further 23 days to review the remaining predicted true-positive data (those with a TM score >28%).

#### Manual curation quality

Table 10 shows the review of our manual curation quality. The intra-agreement rate was 0.84, while the inter-agreement rate was 0.9 to give an overall agreement rate of 0.87. Cohen’s Kappa coefficient (56) showed a moderate intra-agreement rate of 0.43, a substantial inter-annotator agreement rate of 0.80 and a substantial overall agreement rate of 0.73. On review of the curation results that were in disagreement, 7 of the 8 new curation results in the intra analysis were correct. Four were caused by incorrect normalization to protein IDs and one by incorrect protein tagging and it is likely that these were identified in the second attempt owing to increased experience in curating pain-related proteins. A further two were attributed to event mismatches. In the inter analysis, 5/5 of the new curation results were correct and the original curation errors were again due to erroneous protein normalization and also more complex interactions that were perhaps more difficult to curate correctly. While this assessment of our manual curation quality showed that our curated results were of a high standard, they also show that it is likely that some of the curated data that have not been reviewed are likely to be incorrect. Therefore, to be sure that the final curated results used in subsequent analyses are entirely accurate, it is important to perform multiple curations.

**Table 10.** Manual curation evaluation

Analysis	TPs before	TPs after	FPs before	FPs after	Agreed	Disagreed	P(A)	P(E)	K
Intra	18	12	32	38	42	8	0.84	57.3	0.427
Inter	27	22	23	28	45	5	0.9	49.5	0.802
Overall	45	34	55	66	87	13	0.87	51.6	0.731

We evaluate the quality of our manual curation using an intra analysis (data quality is evaluated by the same curator), an inter analysis (data quality originally curated by a different curator is evaluated) and these two are combined to show an overall evaluation of our manual curation. We present the number of true positives (TPs) and false positives (FPs) in the original curation (before) and the new curation results (after). Results that were the same were marked as ‘Agreed’ and those that were different, ‘Disagreed’. The absolute agreement, P(A), was calculated from the proportion of agreement (agreed/disagreed). Cohen’s Kappa coefficient (K) was calculated from the proportion of agreement, corrected for expected agreement by chance [P(E)], such that K = {[P(A) – P(E)]/[1-P(E)]}.

## Conclusions

In this study we have demonstrated that a pain-specific contextual molecular interaction database can be created using TM to rapidly generate content and support manual curation to confirm its accuracy. The whole process of building the pain-relevant corpus, extracting and contextualizing the interactions and curating the data took just 2 months, which is in contrast to a typical fully manual procedure that may take years. We have used the existing state-of-the-art in TM methods to generate the core data used in our curation (e.g. corpus generation using LINNAEUS and event chains and context taken from BioContext). Therefore, the approach used in this study is not limited to the pain domain and would potentially suit many other biomedical fields that consider molecular interactions a focal point of the research. For example, the approach could be repeated for another topic by applying a relevant dictionary to generate a corpus in the same way as for pain and using this as a basis for data extraction and curation. To facilitate such instantiations of our approach in other fields, we have therefore provided a full list of methods used in this study on wiki-pain.org/downloads.

As well as the existing TM methods and data used in this study we have also proposed a (i) new method for scoring documents for their relevance to pain and any individual concepts; (ii) new methods for determining the relevance of an event chain to pain or disease terms and (iii) a novel sentence-based mutation-protein linking extension to MutationFinder. Furthermore, wiki-pain.org is the first extensive pain-specific molecular interaction database that researchers can use to explore context specific pain data extracted from the literature.

In the future, we wish to continue curating the grouped molecular interactions for pain and to expand this curation process to each individual context to ensure that all of our data is accurate. We then plan to investigate more closely the biological implications of the data. For example, it would be interesting to compare and contrast the most connected and frequently occurring proteins between different pain-related disorders and anatomical regions. Furthermore, our procedure has been carefully designed so that additional context can be built into our database and adding aspects such as chemical interactions will be considered.

## Supplementary Data

Supplementary data are available at *Database* Online.

Supplementary Data
