# Supplementary material for: Cataloging the biomedical world of pain through semi-automated curation of molecular interactions
Source: Database (Oxford). 2013 May 23;2013:bat033. doi: 10.1093/database/bat033 (PMC3662864; doi:10.1093/database/bat033)
Supplement: Supplementary Data [file supp_2013_bat033_index.html]

Supplementary Data 

# Cataloging the biomedical world of pain through semi-automated curation of molecular interactions

## Supplementary Data

files

**Files in this Data Supplement:**

- Supplementary Data - zip file
