# Supplementary material for: Cataloging the biomedical world of pain through semi-automated curation of molecular interactions
Source: Database (Oxford). 2013 May 23;2013:bat033. doi: 10.1093/database/bat033 (PMC3662864; doi:10.1093/database/bat033)

**Jamieson et al. Cataloging the biomedical world of pain through semi-automated curation of molecular interactions.**

**Supplementary File 3**

Each event chain (E) and term (T) has it’s own relevance score (R). This score is calculated based on the sections that T and E are denoted in and if appropriate the distance (D) between E and T, and the order (O) in which E and T are presented in the document. O=1 if T occurs before E in the text. O=2 if E occurs before T. D is calculated by deducing the number of characters between E and T. Each type of calculation for R based on the section of E and T is presented below.

**Overlap (E, T)** – the position of E and T overlaps

- R=100

**Sentence (T, E)** – T and E are presented in the same sentence

- R = 100 - (D * 0.02 * O)
- If R <75, R=75

**Title (T, E)** – T and E are both present in the title

- R = 100 – (D * 0.02 * O)
- If R<75, R=75

**Abstract (T), Title (E)** – T is presented in the abstract, E is presented in the title

- R= 100 - (D * 0.02 * O)
- If R<25, R=25

**Abstract (T, E) –** T and E are both presented in the abstract

- R= 100 - (D * 0.04 * O)
- If R<25, R=25

**Body (T, E)** – T and E are both presented in the body

- R= 100 - (D * 0.04 * O)
- If R<5, R=5

**Title (T), Abstract (E)** – T is presented in the title, E is presented in the abstract

- R=75

**Title (T), Body (E)** – T is presented in the title, E is presented in the body

- R=50

**MeSH (T), Title (E)** – T is a MeSH term, E is presented in the title

- R=50

**MeSH (T), Abstract (E)** – T is a MeSH term, E is presented in the abstract

- R=25

**Mesh (T), Body (E)** **–** T is a MeSH term, E is presented in the dody

- R=10

**Abstract (T), Body (E)** – T is presented in the abstract, E is presented in the body

- R=5

**Body (T), Title (E)** - T is presented in the body, E is presented in the title

- R=5

**Body (T), Abstract (E)** – T is presented in the body, E is presented in the abstract

- R=5

The table below provides some examples of R values for terms and the information used to calculate them.

| Term | Distance between T and E (chars) | Section of T to E (T, E) | T is before or after E | R |
| --- | --- | --- | --- | --- |
| Spinal cord | 55 | Body, body | after | 95.6 |
| Unstable angina | 360 | Abstract, abstract | after | 71.2 |
| Serotonin | 0 | sentence | overlap | 100 |
| Dopamine | 1,407 | Title, abstract | before | 75 |
| Pain | 1,014 | Abstract, abstract | before | 59.44 |
| Inflammatory | 1,399 | Body, body | before | 44.04 |
| Depression | 62,655 | Body, body | after | 5 |
| Pain | 1,300 | Title, body | before | 50 |
| Chronic pancreatitis | 1,128 | Abstract, body | before | 5 |
| Anesthesia | 2,268 | Body, body | before | 9.28 |

Overall Pain Relevance Score

The overall pain relevance score (p) for any given E is calculated using all pain Ts that have R>50 for E (N). Such that,


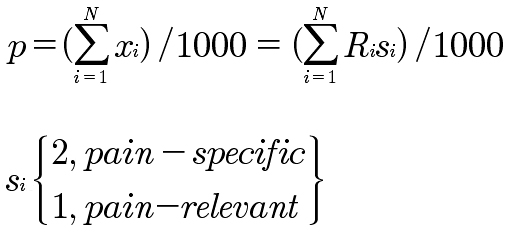


Overall Disease Relevance Score

A similar calculation is used to produce an overall disease relevance score (*p*) for any given E using the disease Ts that have an R>50 (*N*).


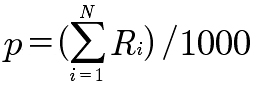

Supplement: Supplementary Data [file supp_bat033_suppl_data.zip › Supplementary File 3.docx]
