# Supplementary material for: Cataloging the biomedical world of pain through semi-automated curation of molecular interactions
Source: Database (Oxford). 2013 May 23;2013:bat033. doi: 10.1093/database/bat033 (PMC3662864; doi:10.1093/database/bat033)
Supplement: Supplementary Data [file supp_bat033_suppl_data.zip › Supplementary File 4.docx]

**Jamieson et al. Cataloging the biomedical world of pain through semi-automated curation of molecular interactions.**

**Supplementary File 4**

The overall pain document relevancy scores are summarized in Figure 4. The analysis of this scoring scheme showed that documents with the MeSH term ‘Pain’ as a major term scored significantly higher than those that had ‘Pain’ as minor MeSH term when using a Wilcoxon/Krustai-Wallis test (Z=-49.326 and p<0.001).


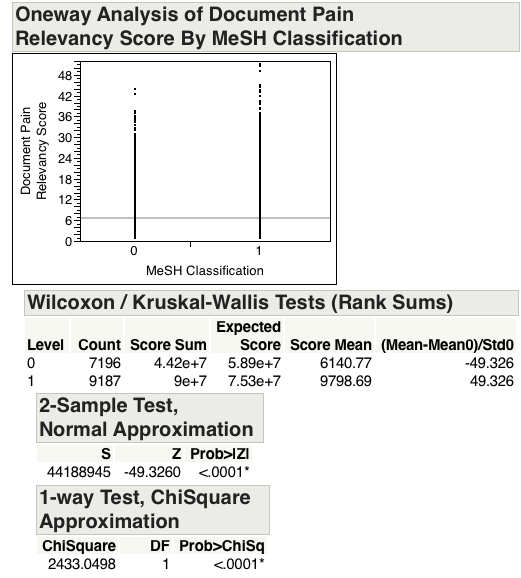


Data was generated using JMP. In the Oneway Analysis of Document Pain Relevancy scores by MeSH classification, 0 is equal to pain terms that are classified as minor and 1 is equal to pain terms that are classified as major on the x axis.
