# Supplementary material for: Cataloging the biomedical world of pain through semi-automated curation of molecular interactions
Source: Database (Oxford). 2013 May 23;2013:bat033. doi: 10.1093/database/bat033 (PMC3662864; doi:10.1093/database/bat033)
Supplement: Supplementary Data [file supp_bat033_suppl_data.zip › Supplementary File 7.docx]

**Jamieson et al. Cataloging the biomedical world of pain through semi-automated curation of molecular interactions.**

**Supplementary File 7**

Having manually curated 4% of the interactions we sought to infer what proportion of the uncurated interactions were likely to be true positives. The TM confidence score for each interaction (deduced by Biocontext) separates the true from false positives well.


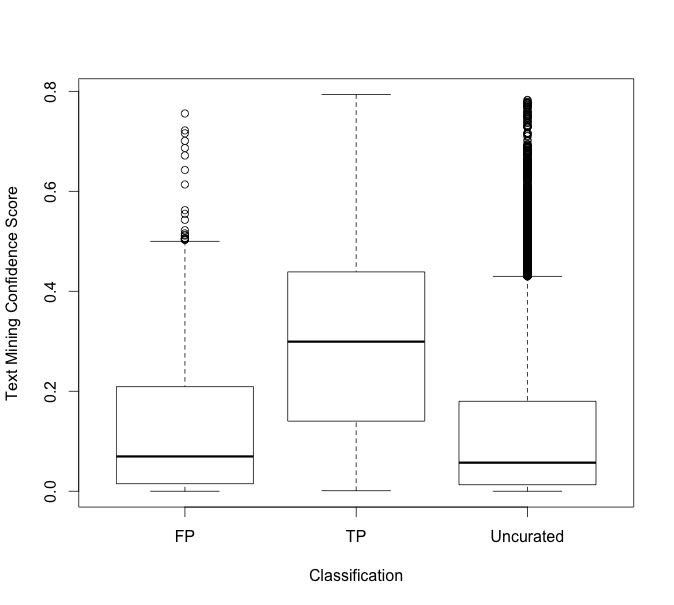


The true and false positive interactions have a mean confidence score of 0.3 and 0.1 respectively and are significantly different (two-tailed students t-test p<0.0001). We therefore fit a generalized linear model (GLM) following a binomial distribution with a logit link function to the confidence scores from the curated data, so that we could assign a probability of being correct to the remaining 36,732 grouped interactions.

As shown in the plot below, conditional density estimates were used to determine the probability of an interaction being a true or false positive conditional on the TM confidence score.


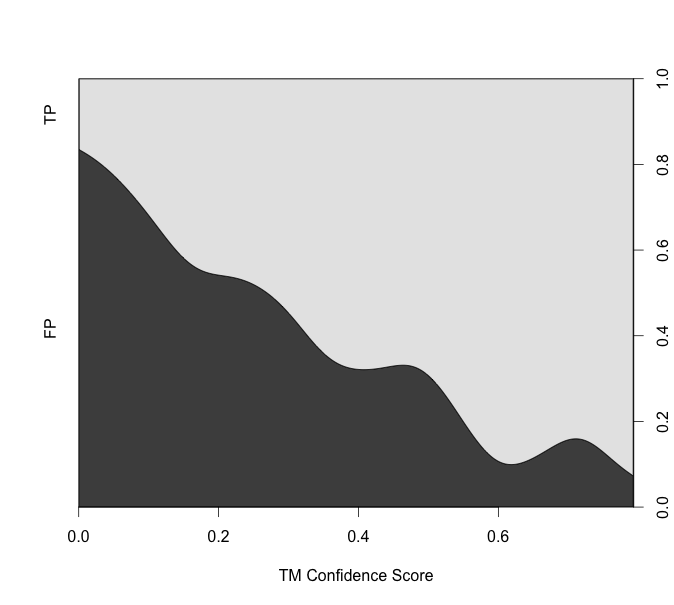


In essence we find that interactions with a TM confidence score >0.28 are likely to be true positives.  Using this measure we predict that 5,816 of the remaining interactions are more likely to be true positives than false positives
