# Supplementary material for: Cataloging the biomedical world of pain through semi-automated curation of molecular interactions
Source: Database (Oxford). 2013 May 23;2013:bat033. doi: 10.1093/database/bat033 (PMC3662864; doi:10.1093/database/bat033)
Supplement: Supplementary Data [file supp_bat033_suppl_data.zip › Supplementary_file1.docx]

**Jamieson et al. Cataloging the biomedical world of pain through semi-automated curation of molecular interactions.**

**Supplementary File 1**

The criteria used to define each pain terms category.

| **Category** | **Criteria** |
| --- | --- |
| Anatomy | A term that refers to an anatomical region. |
| Condition | A condition that may later lead to the development of a disorder. |
| Disorder | A biomedical disorder usually associated with a specific disease. |
| Drug | A drug name. |
| Drug Class | A term that refers to the class of a drug. |
| Family | A term that signifies a group or class of molecules. |
| Molecule | Specific molecule names. |
| Other | Pain terms that do not fit the other categories. |
| Pain type | A descriptive term for a type of pain. |
| Process | A biological process. |
| Response | A term that signifies a measurable biological attribute. |
| Treatment | A term that refers to some form of medical treatment. |
