# Supplementary material for: Cataloging the biomedical world of pain through semi-automated curation of molecular interactions
Source: Database (Oxford). 2013 May 23;2013:bat033. doi: 10.1093/database/bat033 (PMC3662864; doi:10.1093/database/bat033)
Supplement: Supplementary Data [file supp_bat033_suppl_data.zip › supp_file_5.docx]

**Jamieson et al. Cataloging the biomedical world of pain through semi-automated curation of molecular interactions.**

**Supplementary File 5**

We manually curated rat, human and mouse molecular events (i.e. a protein and a linked event) and any linked instances of negation and speculation in each of the 5 documents to create a gold standard dataset (GS). We assessed precision, recall and F score for TM by using all unique event chains from GS involving mouse, rat and human proteins, and excluding self-interactions, against of gold standard of five full text documents. These results are summarized in the table below.

| **Event Chain Type** | **True Positives** | **False Positives** | **False Negatives** | **Precision (%)** | **Recall (%)** | **F Score (%)** |
| --- | --- | --- | --- | --- | --- | --- |
| Single events | 143 | 264 | 29 | 35.13 | 83.14 | 49.40 |
| Molecular interactions | 66 | 120 | 101 | 35.48 | 39.52 | 37.39 |
| More than two proteins | 1 | 9 | 19 | 10.00 | 5.00 | 6.67 |
| All event chains | 210 | 393 | 149 | 34.83 | 58.50 | 43.66 |

TM data is divided into a number of different event chain sub-types, including single events (event chains involving 1 protein), molecular interactions (events involving two proteins) and event chains involving ‘more than two’ proteins. True positives, false positives and false negatives were determined from a gold standard comparison dataset.

The overall precision, recall and F score were 35%, 58% and 44% respectively where the full event chain documented in the text had been extracted perfectly by TM. We noted similar precision for single events and molecular interactions, however, much lower recall in molecular interactions than single events. In our analysis of the common causes of falsely reported data we noted event mismatches often occurring in complex sentences where numerous event triggers and proteins were present. We also noted erroneous conversion of special characters in proteins such as alpha or gamma, which subsequently caused incorrect sentence splitting, preventing linked events and proteins from being extracted correctly in the sentence restricted TM software used by Biocontext.
