# Supplementary material for: Cataloging the biomedical world of pain through semi-automated curation of molecular interactions
Source: Database (Oxford). 2013 May 23;2013:bat033. doi: 10.1093/database/bat033 (PMC3662864; doi:10.1093/database/bat033)
Supplement: Supplementary Data [file supp_bat033_suppl_data.zip › supp_file_6.docx]

**Jamieson et al. Cataloging the biomedical world of pain through semi-automated curation of molecular interactions.**

**Supplementary File 6**

We performed a comparison of event chains extracted by TM in comparison to interactions present in the existing manually curated iRefIndex database to determine the novelty of the interactions we were extracting. As iRefIndex is not explicitly pain relevant we compared interactions extracted from the same documents in each dataset. Note, we were only able to use documents that were PMC open access, as iRefIndex contains molecular interactions extracted from full text not available to TM. Finally we limited our comparison to documents only containing human, mouse and rat protein interactions and excluding self-interactions in iRefIndex, as these were the same criteria used in our final curation step of this study. Overall there were 9 documents that met these criteria. We then randomly selected 5 of these documents to be used for closer analysis between the two datasets. To match our TM data to the iRefIndex data format we used only event chains containing two protein participants and then removed any linking events. We also limited the comparison between the two datasets to only molecular interactions involving human, mouse or rat proteins (normalized to an Entrez Gene ID) and no self-interactions, as this reflected our curation criteria used later in the study.

In the comparison of manually curated data from iRefIndex to TM from 5 full text documents, iRefIndex contained 281 unique protein pairs in contrast to 112 in TM, with only 21 pairs present in both datasets. To determine the reason for this disparity between datasets we manually curated rat, human and mouse molecular events (i.e. a protein and a linked event) and any linked instances of negation and speculation in each of the 5 documents to create a gold standard dataset (GS). GS, contained 172 unique single events, 167 unique molecular interactions (from 97 different protein pairs) and 20 unique event chains involving more than two proteins. The table below summarises the data present in iRefIndex, GS and TM (true positives only) for these documents.

| **Dataset** | **Single Events** | **Molecular Interactions** | **Events chains with more than 2 molecules** |
| --- | --- | --- | --- |
| iRefIndex | N/A | 281 protein pairs | N/A |
| TM (true positive data) | 143 | 66 (44 protein pairs) | 1 |
| GS | 172 | 167 (97 protein pairs) | 20 |

Data present in 5 full text documents from iRefIndex, TM true positives and GS. We report data from each dataset for single events, molecular interactions and event chains that contain more than two molecules. iRefIndex only contains protein pairs and does not contain any data for single events or event chains with more than two molecules. We provide total unique molecular interactions for TM and GS with linking events and also total numbers of unique protein pairs in brackets.

Interestingly, from the 97 protein pairs in GS only 28 were included in iRefIndex, leaving 69 GS protein pairs unaccounted for. While the 69 protein pairs not present in the iRefIndex dataset would appear to be false negatives it is likely that the curation criteria used by the contributing databases in iRefIndex differed from ours during GS curation. For example, molecular interactions cited from past studies in a paper are generally not curated in iRefIndex, whereas they were included in the GS dataset. However, we noted that over two thirds of the 69 protein pairs not in iRefIndex were reported directly in the 5 documents analysed. Furthermore, there were 253 iRefIndex molecular interactions not reported in GS and these were determined to have been extracted from supplementary files and tables outside of the main text and as such were not curated into GS - these would also not be available to TM. Also, some of the GS event chains containing more than two proteins were represented as molecular interactions in the iRefIndex dataset.
