# Supplementary material for: Cataloging the biomedical world of pain through semi-automated curation of molecular interactions
Source: Database (Oxford). 2013 May 23;2013:bat033. doi: 10.1093/database/bat033 (PMC3662864; doi:10.1093/database/bat033)
Supplement: Supplementary Data [file supp_bat033_suppl_data.zip › supplementary file 2.docx]

**Jamieson et al. Cataloging the biomedical world of pain through semi-automated curation of molecular interactions.**

**Supplementary File 2**

Extracted even chains are categorized with the GENIA ontology into the 9 categories shown in the table.

| Event | Themes Possible | Causes Possible |
| --- | --- | --- |
| Gene expression | 1 protein | A regulatory event |
| Transcription | 1 protein | A regulatory event |
| Protein catabolism | 1 protein | A regulatory event |
| Phosphorylation | 1 protein | A regulatory event |
| Localisation | 1 protein | A regulatory event |
| Binding | 1-2 protein(s) | A regulatory event |
| Regulation | 1 protein or event | 1 protein or event |
| Positive regulation | 1 protein or event | 1 protein or event |
| Negative Regulation | 1 protein or event | 1 protein or event |

Some examples of the possible combinations of proteins and events that can form the themes and causes of each event in an event chain are given below for each of the 9 event types.

Gene Expression

- **Gene expression** of protein A
- Regulation of **Gene expression** of protein A
- Protein B Positive Regulation of **Gene expression** of protein A

Transcription

- **Transcription** of protein A
- Positive Regulation of **Transcription** of protein A
- Protein B Negative Regulation of **Transcription** of protein A

Protein Catabolism

- **Protein catabolism** of protein A
- Protein B Regulation of **Protein Catabolism** of Protein A
- Negative regulation of **Protein catabolism** of protein A

Phosphorylation

- **Phosphorylation** of protein A
- Positive regulation of **Phosphorylation** of Protein A
- Protein B Positive regulation of **Phosphorylation** of Protein A

Localisation

- **Localisation** of protein A
- Regulation of **Localisation** of protein A
- Protein B Positive Regulation of **Localisation** of protein A

Binding

- **Binding** of protein A
- **Binding** of protein A and protein B
- Regulation of **Binding** of protein A
- Protein C Regulation of **Binding** of protein A and protein B

Regulation

- **Regulation** of protein A
- **Regulation** of Gene Expression of Protein A
- Protein B **Regulation** of protein A
- Protein B Negative regulation of **Regulation** of Transcription of Protein A
- Protein D Negative Regulation of Protein C **Regulation** of Binding of Protein B and Protein A

Negative Regulation

- **Negative Regulation** of Protein A
- **Negative Regulation** of Positive regulation of Protein A
- Protein B **Negative Regulation** of Protein A
- Regulation of **Negative Regulation** of Protein A
- Protein C Positive Regulation of Protein B **Negative Regulation** of Protein A

Positive Regulation

- **Positive regulation** of Protein A
- **Positive regulation** of Binding of Protein A
- Protein B **Positive regulation** of Protein A
- Protein B Negative Regulation of **Positive Regulation** of Protein catabolism of Protein A
- Regulation of Protein B **Positive regulation** of Protein A
